# How to find and use validated blood pressure measuring devices

**DOI:** 10.1038/s41371-022-00718-5

**Published:** 2022-07-01

**Authors:** Dean S. Picone, Raj Padwal, George S. Stergiou, Jordana B. Cohen, Richard J. McManus, Siegfried Eckert, Kei Asayama, Neil Atkins, Michael Rakotz, Cintia Lombardi, Tammy M. Brady, James E. Sharman

**Affiliations:** 1 Menzies Institute for Medical Research, University of Tasmania, Hobart, Australia; 2 Department of Medicine, University of Alberta, Edmonton, AB Canada; 3 Hypertension Center STRIDE-7, National and Kapodistrian University of Athens, School of Medicine, Department of Medicine, Sotiria Hospital, Athens Greece; 4 Renal-Electrolyte and Hypertension Division, Department of Medicine and Department of Biostatistics, Epidemiology, and Informatics, Perelman School of Medicine, University of Pennsylvania, Philadelphia, PA USA; 5 Nuffield Department of Primary Care Health Sciences, University of Oxford, Oxford, UK; 6 German Hypertension League, Heidelberg, Germany; 7 Department of Hygiene and Public Health, Teikyo University School of Medicine, Tokyo, Japan; 8 Studies Coordinating Centre, Research Unit Hypertension and Cardiovascular Epidemiology, KU Leuven Department of Cardiovascular Sciences, University of Leuven, Leuven, Belgium; 9 Medaval Ltd., Unit 107, SBC, Serpentine Ave., Ballsbridge, Dublin D04 H522 Ireland; 10 American Medical Association, Chicago, IL USA; 11 Department of Non-Communicable Diseases and Mental Health. Pan American Health Organization, Washington, DC USA; 12 Department of Pediatrics, Division of Nephrology, Johns Hopkins University School of Medicine, Baltimore, MD USA

**Keywords:** Risk factors, Diagnosis

## Abstract

Clinically validated, automated arm-cuff blood pressure measuring devices (BPMDs) are recommended for BP measurement. However, most BPMDs available for purchase by consumers globally are not properly validated. This is a problem because non-validated BPMDs are less accurate and precise than validated ones, and therefore if used clinically could lead to misdiagnosis and mismanagement of BP. In response to this problem, several validated device lists have been developed, which can be used by clinicians and consumers to identify devices that have passed clinical validation testing. The purpose of this review is to describe the resources that are available for finding validated BPMDs in different world regions, to identify the differences between validated device lists, and describe current gaps and challenges. How to use validated BPMDs properly is also summarised.

## Introduction

It is difficult to measure blood pressure (BP) accurately using the traditional manual auscultatory method. Automated, ‘press-button’ BP measuring devices (BPMDs) have simplified the measurement process greatly. Automated BPMDs eliminate most user-related errors in BP measurement that are common to manual BP [1, 2]. However, automated BPMDs are not without limitation [1], and should be tested rigorously for clinical accuracy and precision, in a standard process known as validation [3]. BPMDs are considered clinically validated if the minimum accuracy and precision requirements of a scientifically accepted protocol are met. BPMDs identified as technically identical to a previously validated BPMD in components related to measurement accuracy and precision (e.g., cuff, transducer, firmware controlling inflation and deflation, signal processing or algorithm to determine BP) are also considered validated. These are often referred to as equivalent or derivative devices.

All clinical validation studies should be conducted independently of the manufacturer to avoid potential conflicts of interest. New clinical validation studies of cuff-based BPMDs should adhere to the recently published ISO standard (ISO 81060-2:2018) [4], also commonly referred to as the ‘universal standard’ [5]. The importance of clinical validation of BPMDs and specifics of the universal standard are detailed elsewhere in this special edition [6], and other literature [3].

Most automated BPMDs available for purchase by consumers globally have not been properly validated for accuracy [7–9]. Potential reasons for low clinical validation rates include the expense and time required to conduct validation studies and that many BPMDs may not meet the valuation criteria [10]. There is also little incentive for manufacturers to validate their BPMDs because most medical device regulators do not require evidence of clinical validation for pre-market clearance [11]. Therefore, pre-market clearance by most regulatory bodies should not be viewed as synonymous with clinical validation for accuracy [12].

This problem has created the need for resources to help clinicians and consumers determine which BPMDs have passed clinical validation testing. The primary resources that have been developed are validated device lists. There are several lists that summarise information from the clinical validation studies of BPMDs and are designed to be accessible to a non-specialist audience [13]. This brief review will provide practical information on how to find validated BPMDs, including up-to-date information about validated device lists. The differences between the validated device lists, gaps and challenges will also be discussed. Finally, practical resources available to guide the use of validated, automated BPMDs will be summarised.

### Practical information on how to find validated BPMDs

Online validated device lists can be used to find validated BPMDs. The lists are developed and maintained by hypertension societies or not-for-profit collaboratives, as well as by one for-profit private organisation (Table 1). Hypertension societies from several countries have developed validated device lists specific to their countries. There are also two general lists, which contain information on BPMDs that are marketed globally. Detailed practical guidance on how to use the validated device lists was published recently as an open-access document [14]. In brief, if there is a country-specific validated device listing available, then consumers within that region should use it in preference to the general lists. This is because some of the BPMDs listed on country-specific lists may not be found on the global ones, some BPMDs on the general lists are not available in specific countries and the available model numbers of devices can differ between countries. The practical guidance document contains step-by-step instructions on using the general lists and the resources are available in 15 languages from https://bit.ly/ResourcesBP.

**Table 1:** Online validated device lists of blood pressure measuring devices that have been tested for accuracy according to best practice scientific protocols.

**Country-specific lists**	
**American Medical Association** https://www.validatebp.org/	
**Overview**	• Manufacturers must submit application for their BPMDs to be considered for addition to the listing.
	• The US VDL has an advisory group, including representatives from numerous organisations, and an independent expert review committee which reviews manufacturer documentation and makes determination regarding listing by consensus. The listing is not dependent on published studies but validation studies must be performed by an independent party.
**Types of BPMDs listed**	Upper-arm and wrist cuff eligible.
	Cuffless wearables not currently considered.
**Validation protocols accepted**	• ISO 81060-2:2018
	• ANSI/AAMI/ISO 81060-2: 2013
	• ANSI/AAMI/ISO 81060-2: 2009
	• ANSI/AAMI SP10: 2002
	• BHS Revised Protocol: 1993
**Procedure for equivalent / derivative BPMDs**	Notarized affidavit stating there are no differences between the new BPMD and validated BPMD in:
	1. BP Algorithm
	2. BP Module Software
	3. Cuff Design, Sizes, or Material
	4. Inflation/Deflation Mechanism or Method
	5. Any other feature that would impact collection of waveform data or calculation of BP result
**Frequency of list updates**	Each independent review committee cycle.
**British and Irish Hypertension Society (BIHS)** https://bihsoc.org/bp-monitors/	
**Overview**	The BIHS Blood Pressure Measurement Working Party review published validation studies against established criteria.
**Types of BPMDs listed**	Upper-arm and wrist cuff included. Alternative devices for measuring BP not currently considered.
**Validation protocols accepted**	• ISO 81060-2:2018
	• ANSI/AAMI/ISO 81060-2: 2013
	• ANSI/AAMI/ISO 81060-2: 2009
	• BHS Revised Protocol: 1993 (minimum B grade)
	• ESH-IP 2010
	• Superseded versions of the above protocols are also accepted.
**Procedure for equivalent / derivative BPMDs**	Manufacturers certify information about equivalence to BIHS regarding:
	• Cuff characteristics i.e. shape, size or materials.
	• Transducer, amplifier and any signal processing carried out prior to digitisation.
	• Cuff inflation
	• Cuff deflation
	• Interpolation
	• Algorithm
**Frequency of list updates**	Several times per year dependent on independent review cycle.
**Hypertension Canada** [36] https://hypertension.ca/bpdevices	
**Overview**	Manufacturers must submit an application for their BPMDs to be considered for addition to the listing.
	Two reviewers without conflict of interest then review the application. Disagreements are resolved by a third reviewer if required.
**Types of BPMDs listed**	• Upper-arm and wrist cuff oscillometric BPMDs are included in the list.
**Validation protocols accepted**	• ISO 81060-2:2018 with 2020 cuff amendment
	• ANSI/AAMI/ISO 81060-2: 2013
	• BHS Revised Protocol: 1993
	• ESH-IP 2010
	• Superseded versions of the above protocols are also accepted.
**Procedure for equivalent / derivative BPMDs**	Notarized affidavit stating there are no differences between the new BPMD and validated BPMD in:
	• cuff
	• transducer, amplifier, digital signal processing
	• inflation/deflation control system including valve, pump and software
	• filtering and signal processing software, including waveform processing and interpolation
	• BP derivation algorithm
**Frequency of list updates**	Dependent upon manufacturer submission of application
**German Hypertension League (Deutsche Hochdruckliga)** https://www.hochdruckliga.de/betroffene/blutdruckmessgeraete	
**Overview**	List gives details of BPMDs that have passed the DHL Quality Seal Protocol, which is based on the DIN EN 540.
	Devices that are validated outside of DHL or with other protocols are not included in the list.*
**Types of BPMDs listed**	• DHL Quality Seal Protocol designed to validate upper-arm cuff and wrist cuff.
	• ISO/FDIS 81060-2
**Validation protocols accepted**	DHL Quality Seal Protocol
**Procedure for equivalent/derivative BPMDs**	Notarized affidavit stating there are no differences between the new BPMD and validated BPMD in:
	1. BP Algorithm
	2. BP Module Software
	3. Cuff Design, Sizes, or Material
	4. Inflation/Deflation Mechanism or Method
	5. Any other feature that would impact collection of waveform data or calculation of BP result
**Frequency of list updates**	Dependent on independent review cycle.
**Japanese Society of Hypertension** https://www.jpnsh.jp/com_ac_wg1.html	
**Overview**	• Information provided by the manufacturers/sales companies is published as is.
	• Design life and maintenance information of each BPMD and its cuff/tube is also disclosed.
	• Information on where/how the end-user can contact the manufacturer.
	• Website disclaimer that the Japanese Society of Hypertension is not involved in the individual contents of the website and do not recommend a specific BPMD.
**Types of BPMDs listed**	Upper-arm cuff only
**Validation protocols accepted**	Lists supplied by manufacturers
**Procedure for equivalent/derivative BPMDs**	Manufacturers provide information about equivalence which is accepted in good faith and disclosed.
**Frequency of list updates**	Annually
**General registries**	
**STRIDE BP** [37] https://stridebp.org/bp-monitors	
**Overview**	• Endorsed by ESH, ISH, WHL and has a detailed governance and international committee structure. Two reviewers from STRIDE-BP independently review each validation study and produce a checklist report. The report is then reviewed by two members of the STRIDE-BP Scientific Advisory Board before devices are listed.
	• Offers accredited e-learning sessions on office, home and ambulatory BP measurement for healthcare professionals
**Types of BPMDs listed**	• Upper-arm and wrist cuff BPMDs are considered, the former classified as “Preferred”. Cuffless wearables not considered.
	• Separate lists of validated devices provided for children and pregnant women.
**Validation protocols accepted**	• ISO 81060-2:2018
	• ANSI/AAMI/ISO 81060-2: 2013
	• ANSI/AAMI SP10: 2002
	• BHS Revised Protocol: 1993
	• ESH-IP 2010
	Superseded versions of the above protocols are also accepted.
	• Upper-arm cuff devices with a published validation study in the last 10 years are classified as “Preferred”
**Procedure for equivalent/derivative BPMDs**	• Submission made by the device manufacturer which is signed by the manufacturer CEO.
	• Submission considered by STRIDE-BP reviewers using the same process as new validation studies.
	• Any differences in the transducer and processing, algorithm, inflation/deflation system or cuff characteristics typically are not regarded as Equivalent, unless the differences can be fully justified. (https://stridebp.org/about-us/principles-for-device-listing)
**Frequency of list updates**	Standard PubMed search every three months
**Medaval** https://medaval.ie/blood-pressure-monitors/	
**Overview**	• Only VDL to list validated and non-validated BPMDs. Over 4000 BPMDs available. Private, for profit organisation that is not affiliated with scientific societies.
**Types of BPMDs listed**	• Upper-arm, wrist cuff, cuffless wearables, other novel devices.
	• Separate lists of devices validated in specific circumstances including pregnancy, children, the elderly, diabetes mellitus and renal disease. Device availability is also indicated.
**Validation protocols accepted**	• ISO 81060-2:2018
	• ANSI/AAMI/ISO 81060-2: 2013
	• ANSI/AAMI SP10: 2002
	• BHS Revised Protocol: 1993
	• ESH-IP 2010
	• DHL Quality Seal Protocol
	Superseded versions of the above protocols are also accepted. Note: BPMDs without evidence of validation are also listed.
**Procedure for equivalent/derivative BPMDs**	Strict definition of equivalence according to the EU MDR criteria for medical devices. (https://medaval.ie/device-equivalence/)
**Frequency of list updates**	Monthly

Wrist cuff BPMDs are not recommended for BP measurement except under circumstances where upper-arm cuff BP measurement is not feasible. *BPMDs* Blood pressure measuring devices, *US VDL* United States Validated Device List, *ISO* International Organization for Standardization, *ANSI* American National Standards Institute, *AAMI* Association for the Advancement of Medical Instrumentation, *BHS* British Hypertension Society, *ESH-IP* European Society of Hypertension International Protocol, *DHL* Deutsche Hochdruckliga (German Hypertension League), *ISH* International Society of Hypertension, *WHL* World Hypertension League, *EU MDR* European Medical Device Regulation. *Only lists monitors that pass the German Hypertension League Quality Seal Protocol [38, 39].

Most BPMDs are validated for use in ‘general populations.’ This refers to subjects aged over 12 years, at least 30% men and 30% women, with a wide range of arm circumferences and BP levels [3, 6]. However, specific validation protocols also exist for individuals with clinical characteristics that are known to affect the accuracy of BP measurement substantially. The current ISO 81060-2:2018 recognises special populations as pregnant women and children aged <12 years. There are specific requirements for the clinical validation of BPMDs in these ‘special populations’. Individuals with arm circumference >42 cm are under consideration for special population status by ISO [2–4]. One complicating factor is that for people arm circumference >42 cm and upper-arm shorter than the minimum cuff width, an appropriate reference measurement method is uncertain [15]. Atrial fibrillation also is a special population where the reference auscultatory method is uncertain and to date there is no agreed validation procedure [3], nevertheless most automated devices tested in this population appear to be inaccurate [16]. Consumers seeking BPMDs for use in special populations should ensure that an appropriate clinical validation study has been performed [3]. Some of the weblinks in Table 1 provide separate lists or filters for clinically validated devices for children and for pregnant women to assist consumers in selecting the right BPMD for their needs.

### Current differences between validated device lists, gaps and challenges

As shown in Table 1, major differences exist between each of the seven validated device lists. Principal sources of difference are the clinical validation protocols accepted by each validated device list and the procedures for accepting equivalent (derivative) BPMDs (Table 1). These differences can create confusion for consumers (e.g., general public, clinicians, health procurement officers, researchers and representatives of national regulatory authorities) when using the different lists to determine the validation status of BPMDs, because one list may identify a BPMD as clinically validated, whereas another list may not. The problem of different clinical validation protocols will ideally be eliminated in the coming years because of the recent development of the ISO ‘universal’ standard validation protocol (ISO 81060-2:2018) [4]. Nevertheless, in the meantime, it is reasonable to accept the older protocols, because BPMDs with evidence of clinical validation measure BP more accurately, and with less variability, than BPMDs without evidence of validation [17–20].

Consensus still needs to be achieved on the definition of equivalent/derivative BPMDs. The levels of evidence currently required from manufacturers for a device to be accepted as equivalent on a validated device lists are varied. These range from acceptance in good faith on the basis of some equivalence information being provided to requiring more stringent notarised affidavits or evidence of adherence to European Union criteria (MEDDEV 2.7/1 rev 4) (Table 1) [21]. Other factors that contribute to confusion and perceived differences regarding equivalence are that manufacturers may not choose to submit equivalence documentation to all validated device lists (leading to being listed on one and not on another), and that identical internal measurement technology may be used in multiple BPMDs with different model numbers or even different manufacturer names, further adding to the challenges in interpreting and verifying accurate devices. In order to resolve the confusion related to equivalence, all stakeholders, including manufacturers should collaborate to find feasible solutions.

Another reason for differences between validated device lists is that some lists include only upper-arm cuff BPMDs, others also include wrist cuff BPMDs, and one list includes any type of device purporting to measure BP, including cuffless wearables. Altogether, it is desirable to achieve greater consistency between validated device lists with respect to protocols and criteria used to determine validation status of BPMDs. This may be achieved through consensus between existing organisations that publish lists or by the implementation of a universally accepted, accredited validated device list to consolidate information (Table 2). Examples of potential consolidated lists are the European Database on Medical Devices and the FDA Unique Device Identification System [22, 23]. However, for these to become internationally accepted as consolidated lists, robust assessment of clinical validation data by experts must be assured.

**Table 2:** Summary of current gaps and challenges related to validated device lists and proposed actions.

Issue	Proposed action
1. Differences in clinical validation protocols accepted by each validated device list and procedures for including (evaluating) equivalent (derivative) BPMDs.	• Consolidate information, ideally through the development of a single, globally accepted validated device list.
	• Consensus to achieve consistent definitions of equivalent/derivative BPMDs
2. Challenges to identify validated devices available in low-middle income countries.	• Development of a single, globally accepted validated device list with country or region-specific filters.
	• Labelling and marketing of BPMDs which is globally consistent (e.g. the same model number used globally for a device)
3. Difficulties in identifying BPMDs clinically validated in special populations and the cuff sizes available for BPMDs.	• Dedicated labelling or pages on validated device lists for BPMDs validated in special populations and a list of cuff sizes available for BPMDs.

*BPMDs* Blood pressure measuring devices.

It may be more challenging to identify clinically validated BPMDs in low-middle income countries than in high-income countries. All five country-specific validated device listings are based in high-income countries (USA, Canada, UK, Japan, Germany), as are the two general lists that are designed for global use (Greece, Ireland). There is anecdotal evidence from some low-middle income countries that finding validated BPMDs is challenging [24]. There are probably multiple reasons for this challenge, including differences between countries in (1) availability of specific BPMDs; (2) labelling and branding, including the absence of model numbers and original equipment manufacturer information; (3) language variations with/without corresponding model number variations and; (4) medical device regulations including importation. These differences mean that current validated device lists may not provide coverage of all validated BPMDs available in all world regions, but nevertheless, are the most user-friendly way to find validated BPMDs.

Weak and fragmented regulatory frameworks around BPMD validation has been identified by the Pan American Health Organisation/World Health Organisation flagship hypertension control program, HEARTS in the Americas [25]. HEARTS is being implemented in 22 countries and >1300 primary health care centres in the Americas and recommendations for strengthening regulatory frameworks to ensure exclusive use of validated BPMDs were recently published [26]. A validated device list that is specific to the region of the Americas is now under development [24, 27]. The problems described likely extend to other low-middle income countries and may also be relevant to high-income countries without their own validated device lists. A solution may be a universally accepted, accredited validated device list with country- or region-specific filters, to allow users to identify the BPMDs available to them locally. In the interim, the development of region-specific validated device lists or expansion of general lists would be a potential way to improve the identification of validated BPMDs in low-middle income countries. Use of globally consistent model numbers for BPMDs (as per the EUDAMED or FDA UDI systems) could allow easier identification of BPMDs that have been clinically validated for accuracy. This is especially important for BPMDs which are identical but are labelled or marketed differently in certain countries or regions.

There are some differences in the types of information provided by validated device lists about BPMDs for use in special populations and use of different cuff sizes. Some validated device lists use specific labelling or separate pages for BPMDs that are validated in special populations. This is an important step to ensure that the individuals who require these BPMDs can easily identify and select a suitably validated device. Some validated device lists provide information about the number and type of cuff sizes that are available for each BPMD. Cuff size and arm circumference range are important device characteristics because the accuracy of BP measurement is contingent on using a correctly sized cuff [1, 28, 29]. Therefore, providing consumers with information about cuff sizes and targeted arm circumference range may be useful as they seek to make an informed choice regarding which BPMD to purchase.

### Practical resources to guide the use of validated, automated BPMDs

Validated BPMDs must be used properly to ensure accurate BP measurements are obtained. There are at least five steps required to obtain accurate BP measurements, which are summarised in a video that was developed by the Welch Center for Prevention, Epidemiology and Clinical Research at the Johns Hopkins Bloomberg School of Public Health [30]. The first step is to ensure the patient has their back supported, legs uncrossed and feet flat on the floor. The second step is to select the correct BP cuff size. A cuff that is too small will give erroneously high measurements, and a cuff that is too large will give erroneously low measurements [28, 29]. The third step is to ensure BP is measured with the cuff on a bare arm, although if this is not practical, a thin layer of clothing is acceptable. Fourth, the arm should be relaxed and supported on a flat surface such as a table, and the cuff must be level with the heart. Finally, the patient should rest before the measurement. The recommended rest period before BP measurement is five minutes. However, the recent “Best Rest” cross-over design randomised controlled trial of 113 participants (55 ± 16 years, 64% women) found that a rest time of zero or two minutes did not give markedly different BP measurements than a five minute rest period for individuals without elevated systolic BP [31]. These findings indicate that shorter rest periods before BP measurement could be acceptable, particularly for BP screening.

Those who are responsible for measuring BP should be skilled in the aforementioned steps required to obtain accurate readings. After initial training, regular refreshers on the correct measurement technique should be mandated [32]. Any training or refresher course should be available to a wide audience and not overly burdensome to allow for broad implementation. With these requirements in mind, a free, online certification course on automated BP measurement was recently developed by the Pan American Health Organization and collaborators including the Lancet Commission on Hypertension Group, the World Hypertension League, Hypertension Canada and Resolve to Save Lives [33]. The course is intended for any person who measures BP and consists of a 12 minute video before a multiple choice quiz. Once the course is completed successfully, a certificate is generated. The certificate is valid for six months before retraining is required. The course is currently available in English, Spanish, Portuguese, French, Italian and Chinese. For home BP measurement, further guidance and other resources such as BP measurement diaries are available from numerous online sources, including the University of Tasmania webpage, accessible at: https://bit.ly/ResourcesBP [34]. The STRIDE-BP website also recently added accredited e-learning modules for office, ambulatory and home BP measurement designed for healthcare professionals [35]. The e-learning content is based on recent guidelines by the European Society of Hypertension and International Society of Hypertension (www.stridebp.org/training).

## Conclusions

In summary, most automated cuff BPMDs available for purchase by consumers globally have not been clinically validated for accuracy. Validated device lists enable consumers to identify accurate BPMDs. However, there are differences between validated device lists due to the validation protocols that are accepted, procedures for inclusion of equivalent devices and the types of BPMDs listed. Further, the different validated device lists may not be relevant across different regions of the world. Altogether, these differences may create confusion for consumers trying to identify clinically validated BPMDs. Other gaps and challenges to optimal use of validated device lists relate to identification of (1) validated BPMDs available for purchase in low-middle income countries, (2) BPMDs validated in special populations, and (3) cuff sizes available for purchase with each device. While selecting a clinically validated BPMD is essential to accurate BP measurement, these devices must also be used properly so that accurate BP measurements are obtained. Skill achievement and maintenance with training and refreshers to ensure that the five essential measurement steps are followed consistently is recommended.

## Data Availability

Not applicable
